# Bio-functional hydrogel coated membranes to decrease T-cell exhaustion in manufacturing of CAR T-cells

**DOI:** 10.3389/fimmu.2025.1513148

**Published:** 2025-06-27

**Authors:** Aida López Ruiz, Eric Slaughter, Kartik Bomb, Samantha L. Swedzinski, Paige J. LeValley, Zaining Yun, Jacob McCoskey, Kara Levine, Jonathan Steen, Joseph Almasian, Aparajita Chatterjee, Christina Carbrello, Dustin S. Chang, Hubaida Fuseini, Yama A. Abassi, Abraham M. Lenhoff, Catherine A. Fromen, April M. Kloxin

**Affiliations:** ^1^ Department of Chemical and Biomolecular Engineering, University of Delaware, Newark, DE, United States; ^2^ Department of Materials Science and Engineering, University of Delaware, Newark, DE, United States; ^3^ EMD Millipore Corporation, Bedford, MA, United States; ^4^ Agilent Technologies, Santa Clara, CA, United States

**Keywords:** biomaterials, hydrogel, T cell, cell therapy, CAR T, bioinspired, coating, ligand

## Abstract

**Introduction:**

Cell therapies have revolutionized cancer treatment, with chimeric antigen receptor (CAR) T-cell therapies at the forefront for the treatment of hematological cancers. However, current manufacturing protocols rely on rapid T-cell activation, which can induce exhaustion and undesirable phenotypes, ultimately reducing the efficacy and persistence of CAR T-cells. Given the importance of T-cell activation as a fundamental step to achieve proliferative phenotypes for cell engineering and expansion, approaches are needed to control activation and increase CAR T-cell quality. To address this need, in this work, we utilized a bioinspired, scalable, tunable platform to direct T-cell activation and decrease exhaustion during CAR T production.

**Methods:**

Hydrogel-coated membranes (HCMs) were designed with different co-stimulatory ligands and a physiologically-relevant substrate modulus inspired by the native microenvironment in which T cells are programmed. Phenotype, activation, and exhaustion markers were used to compare T cells cultured with HCMs or industry standard TransAct. Next, transduction with a CD19 CAR lentivirus was performed, and the killing potential of the resulting CAR T product was evaluated using an *in vitro* cytolysis model.

**Results:**

With this controlled and well-defined system, we hypothesized that a combination of ligands inspired by antigen-presenting cells would promote desired T-cell phenotypes with reduced exhaustion and thereby improved killing efficacy. We found memory phenotypes, minimal exhaustion, and similar activation profiles with HCMs. Additionally, increased T-cell transduction and decreased exhaustion for the CAR T population were observed with HCMs. Further, the killing potential of the resulting CAR T product was evaluated, finding improved *in vitro* cytolysis of target cells with lower variability with HCMs.

**Discussion:**

These results demonstrate the importance of lower T-cell exhaustion in CAR T manufacturing and present significant opportunities to modulate T-cell phenotypes for cell therapy applications using engineered bioinspired materials that display combinations of co-stimulatory molecules.

## 1 Introduction

Cell therapies have emerged as a critical tool in the treatment of cancer (1–3). In particular, chimeric antigen receptor (CAR) T-cells have gained importance as a targeted cancer therapy, where T-cell lymphocytes from humans are engineered to express a CAR protein to enable the recognition and killing of cancer cells (4, 5). Indeed, CAR T therapies have become a pivotal treatment for hematological cancers, with a remission rate of 40%-90% (5, 6). Existing manufacturing protocols require isolation of T cells from the donors followed by *ex vivo* activation, transduction, expansion, and autologous infusion of the modified cells (7, 8). However, high variability of CAR T function between donors and the overall scalability of the process are two contributing factors hindering broader adoption of this treatment paradigm (9, 10). Innovative approaches are needed for controlling T-cell quality during CAR T production, from T-cell activation and transduction to killing efficacy.

Activation of T cells is a fundamental step to achieve proliferative and functional T-cell phenotypes (11). Existing techniques aim to promote a rapid activation that drives robust cell proliferation; however, this approach can lead to a large fraction of exhausted CAR T-cells, limiting their efficacy (9, 12). Current industry standards for T-cell activation (e.g., TransAct, Dynabeads) are based on synthetic particles that mimic the signals of antigen-presenting cells (APCs) by presenting antibody fragments of anti-CD3 and anti-CD28 to achieve T-cell activation and expansion through immunomodulation (13–15). While anti-CD3 and anti-CD28 are the most common ligands to initiate activation (16), the fast activation they promote can lead to T-cell exhaustion (17, 18). Promotion of desired phenotypes during activation and transduction also is important for product efficacy. T cells are broadly categorized as either *i)* naïve (Tn) or *ii)* effector (Teff) before activation and either *iii)* central memory (Tcm) or *iv)* effector memory (Tem) after activation (19, 20). Notably, central memory phenotypes (Tcm) have greater antitumor effects and proliferation compared to effector memory phenotypes (Tem) (21, 22). Further, naïve and stem-like populations have been found to enhance overall CAR T performance (19, 20). Taken together, CAR T production processes are needed that not only promote activation, but also promote desired phenotypes with limited exhaustion for increased efficacy and persistence of CAR T treatments.

Engineering the microenvironment of T cells during the CAR T manufacturing process, inspired by native T-cell programming in the human body, affords opportunities for controlling CAR T quality and efficacy. T cells are natively programmed in the lymphoid organs by antigen-presenting cells (APCs) (23). Scaffolds and microcarriers that mimic this microenvironment during *ex vivo* T cell culture have been shown effective at promoting T-cell activation and expansion (24–28). For example, porous micro-rods functionalized with lipid bilayers have been used as scaffolds for T-cell stimulation (25), where the density of anti-CD3/anti-CD28 displayed can been tuned for personalizing the level of stimulation on an individual-specific basis (29). We recently have established an approach for integrating bioinspired substrates, functionalized hydrogel-coated membranes (HCMs), into a scalable flow-based membrane device, controlling the microenvironment of T cells while enhancing their transduction through lentiviral concentration polarization (30). These versatile and tunable materials have the potential to further promote activation and expansion of T cells while controlling their phenotype and exhaustion in a well-defined environment by the addition of co-stimulatory ligands targeting specific T-cell receptors.

**Figure 1:**
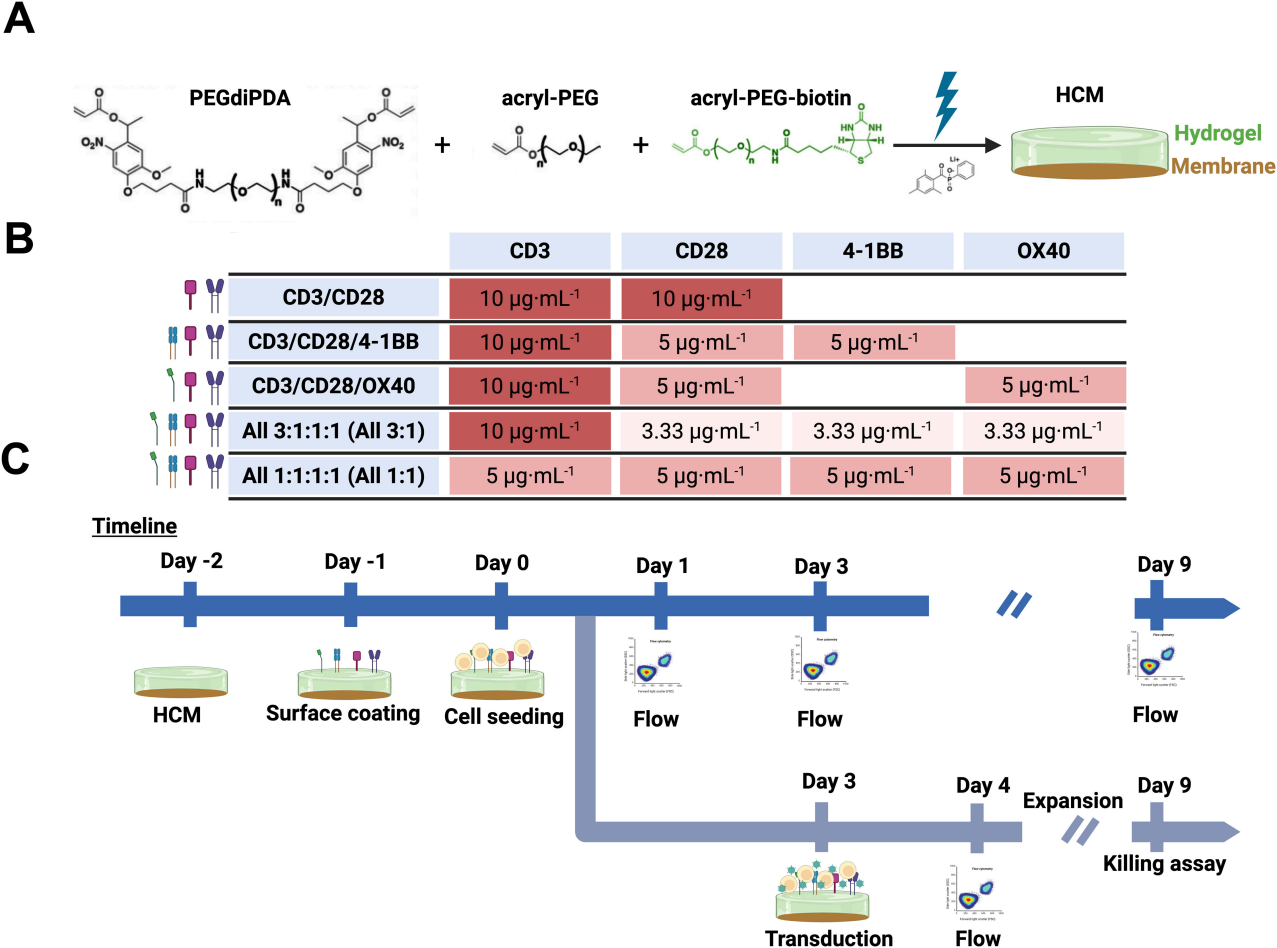
**(A)** Schematic representing HCM formation using PEGdiPDA (black), acryl-PEG (black) and acryl-PEG-biotin (green) to form a hydrogel coating (green) on top of the regenerated cellulose membrane (brown), creating an HCM with physiologically-relevant mechanical properties that can be functionalized with ligands of interest in addition to integration within devices. **(B)** Table representing concentration of antibodies used in each HCM that are color coded by concentration: dark red is 10 μg·mL^-1^ of antibody, medium red is 5 μg·mL^-1^ of antibody, and light red is 3.33 μg·mL^-1^ of antibody. Every combination had a total concentration of 20 μg·mL^-1^ of antibodies to be able to compare between groups. Specifically, for hypothesis testing, the total 20 μg·mL^-1^ of ligand per condition was achieved with 10 μg·mL^-1^ anti-CD3 with 10 μg·mL^-1^ total of co-stimulatory molecule(s) *i)* anti-CD28, *ii)* 1:1 anti-CD28/4-1BB, *iii)* 1:1 anti-CD28/OX40, or *iv)* 1:1:1 anti-CD28/4-1BB/OX40 (All 3:1), as well as *v)* 20 μg·mL^-1^ total with equal amounts of all ligands (1:1:1:1 anti-CD3/CD28/4-1BB/OX40 All 1:1). **(C)** Timeline of activation and transduction (top) or CAR T killing assay (bottom) experiments performed. Specifically, the timepoints selected for the study were day 1, day 3, and day 9 based on their importance for characterization of *i)* initial cellular states (day 1), *ii)* upon activation and the day of transduction (day 3), and *iii)* upon final production of CAR T products (day 9). Created with Biorender.

In this work, we exploit the modular, well-defined bio-inspired hydrogel platform to assess the role of ligand presentation in T-cell activation and expansion. The HCM platform is based on poly(ethylene glycol) diacrylate (PEG-diPhotodegradableAcrylate, PEGdiPDA) hydrogel that integrates acryl-PEG-biotin pendant groups to enable functionalization with a range of co-stimulatory molecules (**Figure 1A**, **Supplementary Figure S1**) (30). Four different co-stimulatory ligands, anti-CD3, anti-CD28, anti-4-1BB, and anti-OX40, were selected as bio-inspired molecules to evaluate their activating effect as artificial APCs. Anti-CD3 and anti-CD28 were used to emulate APC mimics used industrially within biomanufacturing workflows (e.g., TransAct, a soluble colloidal polymeric nanomatrix that presents anti-CD3 and anti-CD28) (16, 31, 32), which promote fast activation and often exhaustion (14). We previously have shown that an HCM functionalized with anti-CD3 and anti-CD28 (1:1, 20 μg·mL^-1^ total) produced activation results comparable to TransAct (30). We hypothesized that adding anti-4-1BB, an enhancer of T-cell survival by suppression of activation induced cell death (33), or anti-OX40, an enhancer of effector T-cell function and survival (34), would enable superior APC mimicry and drive creation of improved functional CAR T-cell products with the modular bio-inspired platform. Further, given the essential nature of anti-CD3 for T-cell activation (16), we hypothesized that maintaining a constant concentration of anti-CD3 across all tested combinations was important (35, 36). To test these hypotheses, we investigated T-cell activation, transduction, and expansion at different ratios of anti-CD3/CD28/4-1BB/OX40 co-stimulatory molecules (**Figure 1B**) over the typical timeframe utilized for industrial CAR T biomanufacturing (**Figure 1C**). Leveraging the tunability of the HCM platform, we found that combinations of co-stimulatory molecules enable a preference for central memory (Tcm) phenotype and reduction of exhaustion when compared to T-cells activated with TransAct. Building from these tunable phenotypes, we found that HCM-generated CD19 CAR T-cells show increased killing efficacy against target (Raji-luc-2) cells as compared to TransAct-generated CAR T-cells. Furthermore, the hydrogel was formed on top of a flat-sheet regenerated cellulose membrane, enabling future integration into a tangential flow filtration device for combining the effects of co-stimulation and flow during the production of CAR T-cells (30). Overall, this work demonstrates the utility of the modular HCM platform for CAR T manufacturing and the overall potential for persistent CAR T therapies.

## 2 Materials and methods

### 2.1 Materials

All specialty reagents used are specified in the methods section as described. All antibodies were purchased from Biolegend (San Diego, CA).

### 2.2 Formation of HCMs

HCMs were created as described in detail in our prior work (30). Briefly, building block PEGdiPDA and photoinitiator lithium phenyl-2,4,6-trimethylbenzoylphosphinate (LAP) were synthesized by established protocols as previously described, and their chemical identity verified with ^1^H NMR (**Supplementary Figures S2**, **S3**) (30). All other building blocks were purchased from commercial sources and used as received. Specifically, hydrogels were formed using PEGdiPDA, PEG-monoacrylate (M_n_ ~ 400 Da from Monomer-Polymer, Ambler, PA), biotin-PEG-acrylate (M_n_ ~ 5 kDa from Biopharma PEG Scientific Inc., Watertown, MA), and LAP. The monomer solution was composed of 4.1 wt% PEGdiPDA, 3.4 wt% PEG-monoacrylate, 1 mg·mL^−1^ biotin-PEG-acrylate, and 3 wt% LAP, a formulation previously shown to result in physiologically-relevant mechanical properties (30). Flat sheet membranes (regenerated cellulose with 300 kDa molecular weight cutoff (MWCO)) were supplied by EMD Millipore Corporation as the base substrate on which the hydrogel was polymerized. Membranes were cut into 2 by 4 cm rectangles prior to coating with the hydrogel. HCMs were prepared by applying 80 μL of monomer solution to the membrane that was seated on a glass draw plate, and the solution was then spread across the membrane using a wet film applicator wire (Gardco, Pompano Beach, FL). Polymerization was achieved by irradiation with filtered, collimated light (λ=400–500 nm, I_0_ = 5 mW·cm^−2^) from an Omnicure (Excelitas Technologies, Mississauga, ON, Canada) for 90 s. The storage modulus was measured using *in situ* rheometry (DHR 30, TA Instruments, New Castle, DE) to ensure achieving an average Young’s modulus on the order of ~ 10 kPa upon polymerization (**Supplementary Figure S4**). After polymerization, the HCMs were cut into rounds using an 8 mm biopsy punch. Rounds were transferred into an untreated 48-well plate and incubated with 70% ethanol for 10 minutes for sterilization. After removing the ethanol, the HCMs were incubated with PBS for 30 minutes; the PBS wash was removed, and HCMs were incubated at room temperature overnight with fresh PBS.

Stability of the hydrogel layer on the membrane was verified by adding acrylate-Rhodamine B (BioChemPEG Scientific Inc., Watertown, MA) to the monomer solution at a concentration of 20 µM, allowing measurement of the height of the hydrogel at physiologically relevant conditions over time using confocal microscopy (Zeiss LSM 800, White Plains, NY) and Image J. Hydrogel rounds were incubated in PBS at 4 °C and at 37 °C (with 5% CO2), and the PBS was replaced every 3 days. The height of four hydrogel rounds stored in each condition was measured at two locations on each gel and three points at each location. Measurements were done at days 1, 3, and 9 (**Supplementary Figure S5**), corresponding to key timepoints in the presented studies and the typical timeframe for manufacturing of CAR T cells.

### 2.3 Functionalization of HCMs

HCMs were functionalized using avidin-biotin chemistry. First, under sterile conditions, HCM rounds were blocked with 1% BSA solution for 1 h at room temperature. After blocking, the BSA solution was removed and 300 μL of 50 μg·mL^-1^ Neutravidin solution was added (NA; ThermoScientific, Waltham, MA) and incubated for 4 h at room temperature. Afterwards, HCMs were washed with PBS twice for 15 min. The biotinylated cell activating antibodies anti-CD3, anti-CD28, anti-41BB, and anti-OX40 were added to the HCM surface and incubated at 4 °C overnight at designated combinations and concentrations (**Figure 1B**). Specifically, the concentrations tested were 1:1 (anti-CD3, anti-CD28), 2:1:1 (anti-CD3, anti-CD28, and anti-41BB), 2:1:1 (anti-CD3, anti-CD28, and anti-OX40), 3:1:1:1 (anti-CD3, anti-CD28, anti-41BB, and anti-OX40) and 1:1:1:1 (anti-CD3, anti-CD28, anti-41BB, and anti-OX40). All the concentrations tested were studied at a total concentration of 20 μg·mL^-1^. For activation experiments, before using the functionalized HCMs, two washes with PBS were performed to remove any unbound antibodies. Finally, HCMs were transferred to a 96-well plate for cell culture.

Substrate modification with antibody at the different concentrations studied was confirmed using anti-CD3 at 5, 10, and 20 μg·mL^-1^, subsequently incubated with a 20 μg·mL^-1^ secondary antibody overnight at 4 °C (AF647 goat anti-mouse IgG1) (**Supplementary Figure S1**). Confirmation of the stability of HCM functionality over 9 days at physiologically relevant conditions was performed by incubating 8 mm HCM rounds functionalized with 20 μg·mL^-1^ of anti-CD3 in complete RPMI (37 °C, 5% CO_2_, humid). Different samples were collected at timepoints of interest (days 1, 3, and 9), washed with PBS (twice), fixed with 4% paraformaldehyde (PFA, 10 min), and then washed and stored in PBS (twice) at 4 °C. After the completion of the experimental time course, all samples were stained with 20 μg·mL^-1^ secondary antibody (AF647 goat anti-mouse IgG1) for 2.5 hours at room temperature (**Supplementary Figure S6**). **Supplementary Figures S1** and **S6** images were taken using a confocal microscope (Zeiss LSM 800, White Plains, NY), and total integrated fluorescence intensity was quantified using FIJI’s measure function.

### 2.4 Isolation of hPBMCs

Human peripheral blood mononuclear cells (hPBMCs) were purchased from Lonza (Basel, Switzerland) from eight different healthy donors. Toward minimizing donor-to-donor variability, donors were selected of the same ethnicity (African American), age range (30–55 years), and non-smoking status (**Supplementary Table S1**). The cells were thawed and incubated for 2 h in RPMI-1640 medium (Corning, Tewksbury, MA) supplemented with 10% heat-inactivated fetal bovine serum (hiFBS) and 1% penicillin-streptomycin (P/S). After 2 h incubation, CD3+ T-cells were isolated using Human Pan T-Cell Isolation Kit (Miltenyi Biotec, Auburn, CA). After isolation, T-cell purity was tested using flow cytometry (FACSAria Fusion, BD Biosciences, San Diego, CA), with anti-CD3-AF700, achieving a purity of >95% (**Supplementary Figure S7**). Isolated T cells were then used for experiments immediately after isolation.

### 2.5 Activation of CD3+ T cells

**Figure 2:**
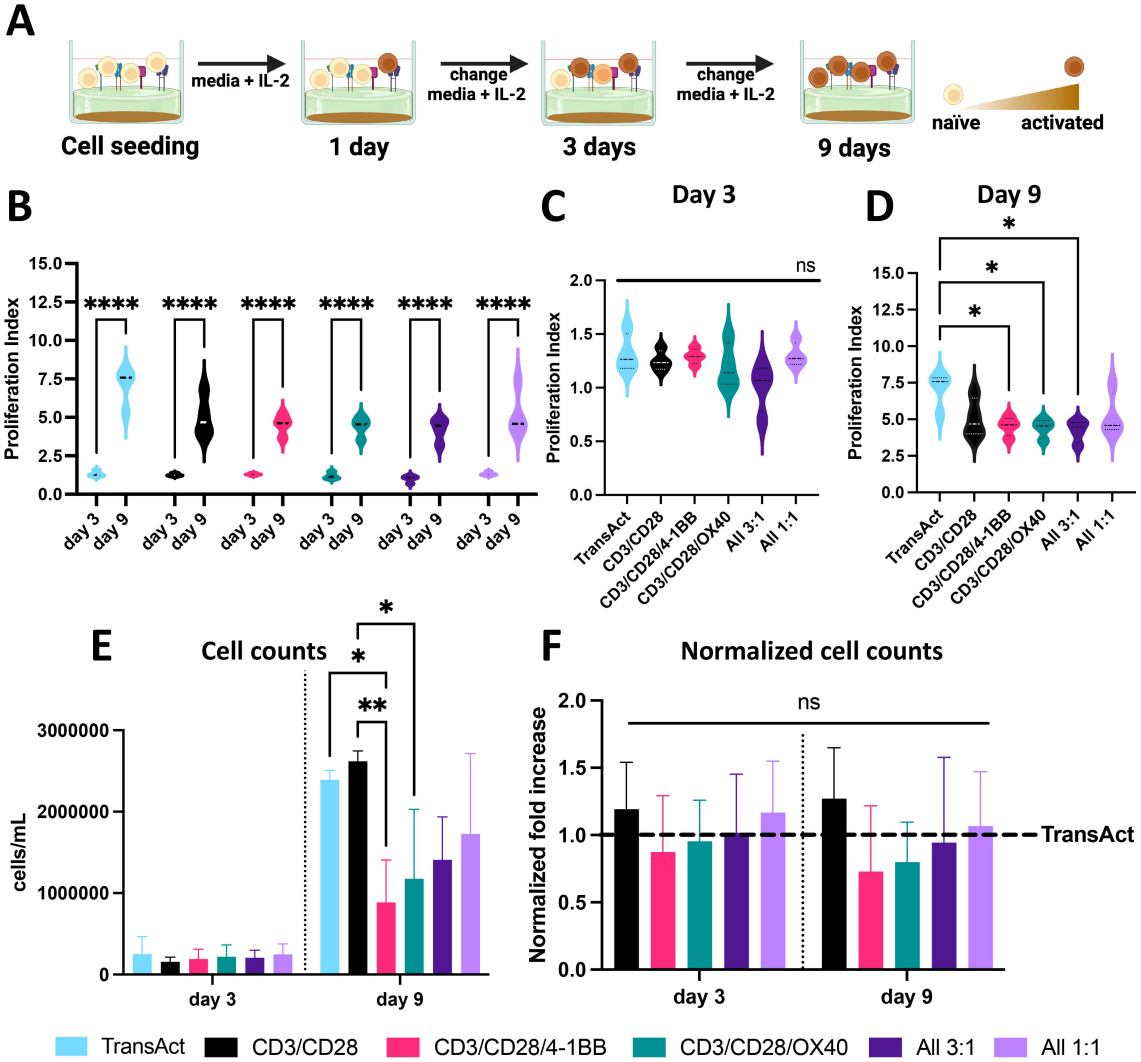
**(A)** Schematic representation of T cell activation using HCMs, showing increase in T cell activation by shift in T cell color from light orange to brown. Created with Biorender. **(B)** Proliferation index of T cells at day 3 and 9 showing statistically significant cell growth for all the groups. **(C)** Proliferation index of all the different groups studied on day 3 showing no significant differences between groups. **(D)** Proliferation index of all the different groups studied on day 9, showing significantly lower PI for 3 groups compared to TransAct. **(E)** Cell counts for all the conditions on day 3 and day 9 (donor 2 was excluded from cell counts due to measurement errors). **(F)** Normalized cell counts to TransAct for each donor on day 3 and day 9 (donor 2 was excluded from normalized cell counts due to measurement errors). Color schemes: TransAct (blue), HMCs, CD3/CD28 (black), CD3/CD28/4-1BB (pink), CD3/CD28/OX40 (green), All 3:1 (dark purple), All 1:1 (light purple). Four different donors with n≥3 for each donor were used as representative sample population. *p< 0.05, **p<0.01 and ****p< 0.0001. Statistical significance was determined using one-way ANOVA with Tukey comparison test between Transact and HCMs, and standard deviation is shown in all the graphs.

Activation of CD3+ T cells was studied after 1, 3, and 9 days of incubation with HCMs or TransAct. Cells were cultured in media with interleukin 2 for the duration of the whole experiment (IL-2, 50 IU·mL^−1^), and media with fresh IL-2 was changed every 3 days (**Figure 2**). For the cells activated with TransAct (Miltenyi Biotec, Basel, Switzerland), cells were incubated at 1:1000 concentration and fresh TransAct was added at every media change with the same concentration.

**Figure 3:**
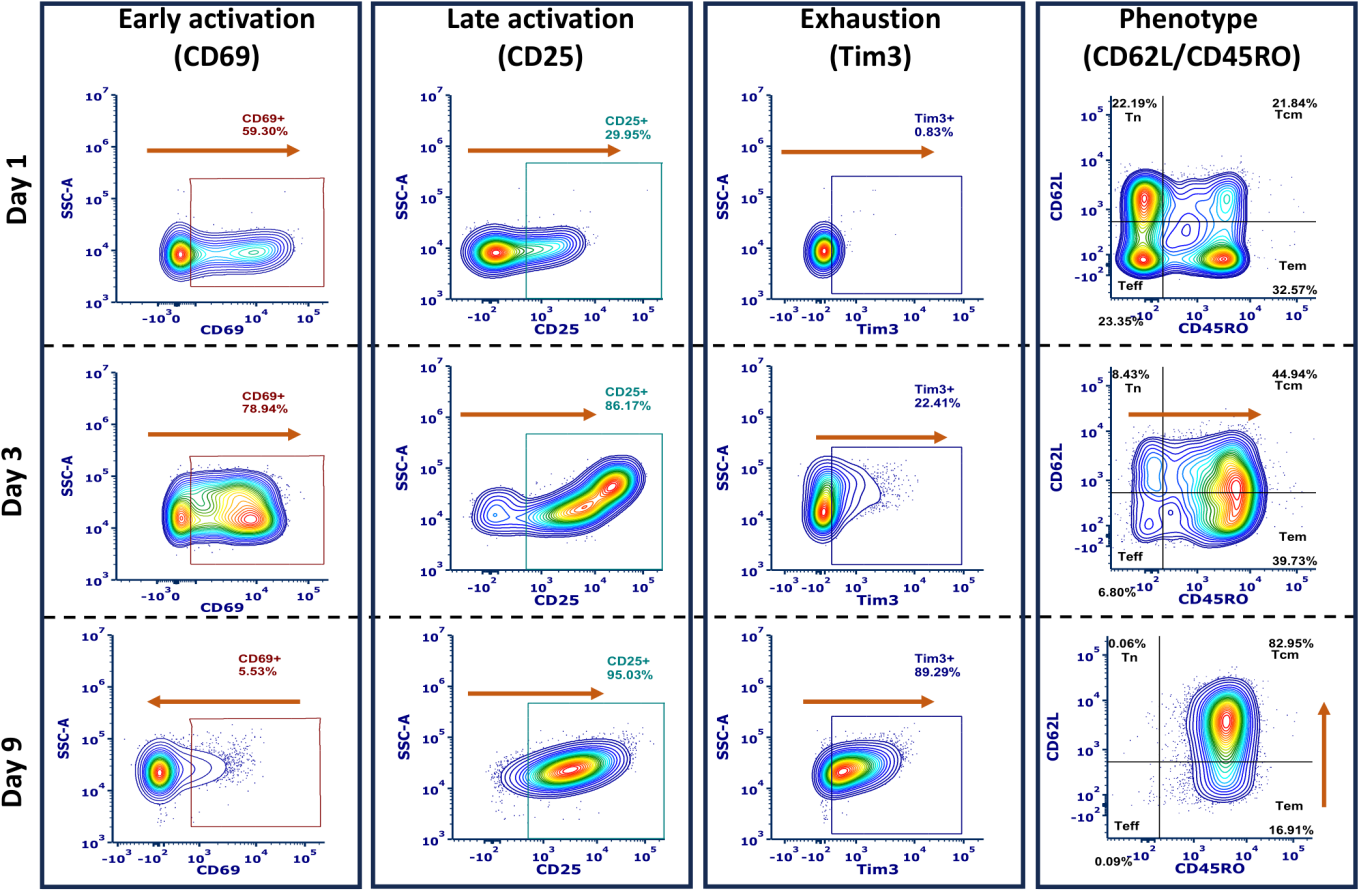
Representative flow cytometry gating scheme for phenotype, activation (early and late), and exhaustion, shown from the same donor activated using TransAct. Flow cytometry profiles are representative of sample changes through the duration of activation experiments for days 1, 3, and 9. Orange arrows represent the increase or decrease of the studied markers through the total length of the experiments.

For flow cytometry preparation, cells were washed twice with PBS and stained with zombie red (Biolegend) for 10 min at room temperature to determine viability. After the first staining step, cells were washed twice with FACS (2% hiFBS in PBS) and stained with the following panel (clones indicated in parentheses): CD25-BV711 (M-A251), CD45RA-BV605 (HI100), PD-1-BV650 (EH12.2H7), Cell trace-BV421, CD62L-FITC (DREG-56), CD4-PE-Cy5 (OKT4), CD69-PE-Cy7 (FN50), CD45RO-PE (UCHL1), CD8-AF700 (SK1), CCR7-APC-Cy7 (G043H7), and Tim3-APC (A18087E). All samples were gated using single-color controls and FMOs (**Figure 3**). Experiments were performed with 4 donors and in triplicates or more for each donor and run on a flow cytometer (FACSAria Fusion, BD Biosciences, San Diego, CA). FCS Express™ software (*De Novo* Software, Pasadena, CA) was used to analyze the data, and all samples were gated using fluorescence minus one and single-color controls.

### 2.6 T-cell proliferation

Cell proliferation was assessed by measuring decreased fluorescence intensity from a Cell Trace-BV421 (Invitrogen, Waltham, MA). Briefly, prior to seeding on substrates for activation studies, cells were stained with 5 μM Cell Trace solution at 37 °C for 20 min in the dark. The remaining dye was quenched using 5 times the volume of media and incubated for 5 min. After quenching the dye, cells were centrifuged at 300 RCF for 10 minutes and resuspended in fresh media for seeding and commencing related cell culture studies. FCS Express™ software was used to determine proliferation profiles at days 3 and 9 by comparing the peak on day 1 to the unstained sample, as reported in **Supplementary Figure S8**. Proliferation index (PI) is defined as the total number of cell divisions divided by the number of cells that underwent division.

### 2.7 T-cell transduction with LentiBrite

Transduction experiments were performed after 3 days on cells activated in a 96-well plate with either HCMs or TransAct. On day 3, cells were counted, and the plate was centrifuged at 200 RCF for 10 minutes to first remove 100 μL of media and then add 100 μL of virus solution. Target cells were transduced with LentiBrite GFP lentivirus (EMD Millipore Corporation, Darmstadt, Germany) at a multiplicity of infection (MOI) of 1. After 24 h, cells were removed from plates and centrifuged at 300 RCF for 5 minutes to remove any unbound virus.

For flow preparation, cells were first stained with zombie yellow (Biolegend) for 10 min at room temperature to determine viability. After the first staining step, cells were washed twice at 300 RCF for 5 minutes with FACS (2% hiFBS in PBS) and stained with the following panel: CD62L-BV421 (DREG-56), CD4-PE-Cy7 (RPA-T4), CD45RO-BV750 (UCHL1), CD8-AF700 (SK1), Tim3-APC-Cy7 (F38-2E2), all purchased from Biolegend. All samples were gated using single-color controls and FMOs. Experiments were performed with 4 donors and in triplicates or more for each donor, run on the flow cytometer (ACEA NovoCyte, Agilent Technologies, Santa Clara, CA), and analyzed with NovoExpress™ software (ACEA NovoCyte, Agilent Technologies, Santa Clara, CA). All samples were gated using fluorescence minus one and single-color controls.

### 2.8 T-cell transduction with CAR 19 lentivirus

Transduction experiments were performed after 3 days of activation on a 96-well plate with HCM combination All 3:1 or TransAct. On day 3, cells were counted, and the plate was centrifuged at 200 RCF for 10 minutes to remove 100 μL of media and then add 100 μL of virus solution. Target cells were transduced with CD19-CAR virus (Vector Biomed, Gaithersburg, MD) at an MOI of 1, 2, and 5. After 24 h, cells were removed from plates and centrifuged at 300 RCF for 5 minutes to remove any unbound virus.

For flow preparation, cells were washed twice with FACS buffer (2% hiFBS in PBS) and incubated with CD19-Fc (R&D Systems, Minneapolis, MN) at room temperature for 10 min. Cells were washed twice at 300 RCF for 5 minutes with FACS buffer to remove unbound protein and stained with anti-human-Fc AF647 (Jackson ImmunoResearch, West Grove, PA) for 20 min. Cells were washed with FACS buffer twice at 300 RCF for 5 minutes and stained with Tim3-BV421 (F38-2E2). Experiments were performed with 3 donors and in triplicate or more for each donor, on a flow cytometer (ACEA NovoCyte, Agilent Technologies, Santa Clara, CA) and analyzed with NovoExpress™ software. All samples were gated using fluorescence minus one and single-color controls.

### 2.9 Killing assays with Raji cells

After transduction with CAR 19 virus, CAR T-cells were expanded for 5 days in RPMI supplemented with 10% heat-inactivated fetal bovine serum (hiFBS), 1% penicillin-streptomycin (P/S), and IL-2, achieving the number of cells needed for killing assays and mirroring timeframes of activation, transduction, and expansion typically used during CAR T-cell production. Raji-Luc2 (CCL-86-LUC2, ATCC, Manassas, VA) were used as target cells for the experiments, and impedance was used to determine cell viability with xCELLigence RTCA MP (Agilent Technologies, Santa Clara, CA). Cell media used for Raji-Luc2 cell culture was ATCC-formulated RPMI-1640 Medium with 10% hiFBS and 1% P/S. RTCA E-plates in 96-well format were used and tethered with 50 μL of tethering dilution (4 μL of Tethering reagent (anti-CD40) + 500 µL of tethering buffer), xCELLigence immunotherapy kit (8100005, Agilent Technologies, Santa Clara, CA). Plates were incubated at 4 °C overnight. Tethering reagent was removed and washed twice with PBS, then 50 μL of Raji media was used to perform a background measurement. Killing assays were set at 1:1 and 3:1 Effector to target ratio, keeping the number of Raji-Luc2 (CCL-86-LUC2, ATCC, Manassas, VA) cells constant at 50,000 cells per well. Raji cells were stained with CellTrace BV421 following the same protocol as indicated for T cells for later image analysis. Volumes of 50 μL of Raji cell suspensions were added to the plate and were equilibrated for 30 minutes, then placed on the xCELLigence overnight for initial impedance measurements. After 24 h, 100 μL of CAR T-cells at different concentrations were added to the Raji cells and left for 30 minutes at room temperature to equilibrate. After 30 minutes, cytolysis reagent was added to the positive control and the plate was attached to the xCELLigence for impedance measurement for 3 days, measured every 15 minutes. Brightfield and DAPI channel (excitation – 359 nm, emission – 457 nm) images were taken using confocal microscopy (Zeiss LSM 800, White Plains, NY) in 24 hours intervals over the 3 days to characterize Raji cell cytolysis (**Supplementary Figure S9**).

### 2.10 Statistics and data normalization

All data represent the mean +/- standard deviation of the mean with the number of biological replicates as “N” and the number of technical replicates as “n”. For unnormalized data, one-way ANOVA with Tukey comparison test between TransAct and HCMs was used to determine statistical differences. For normalized data, a one-sample Wilcoxon test was used. Significance is represented as * p< 0.05, ** p<0.01, *** p < 0.001, and **** p< 0.0001.

**Figure 4:**
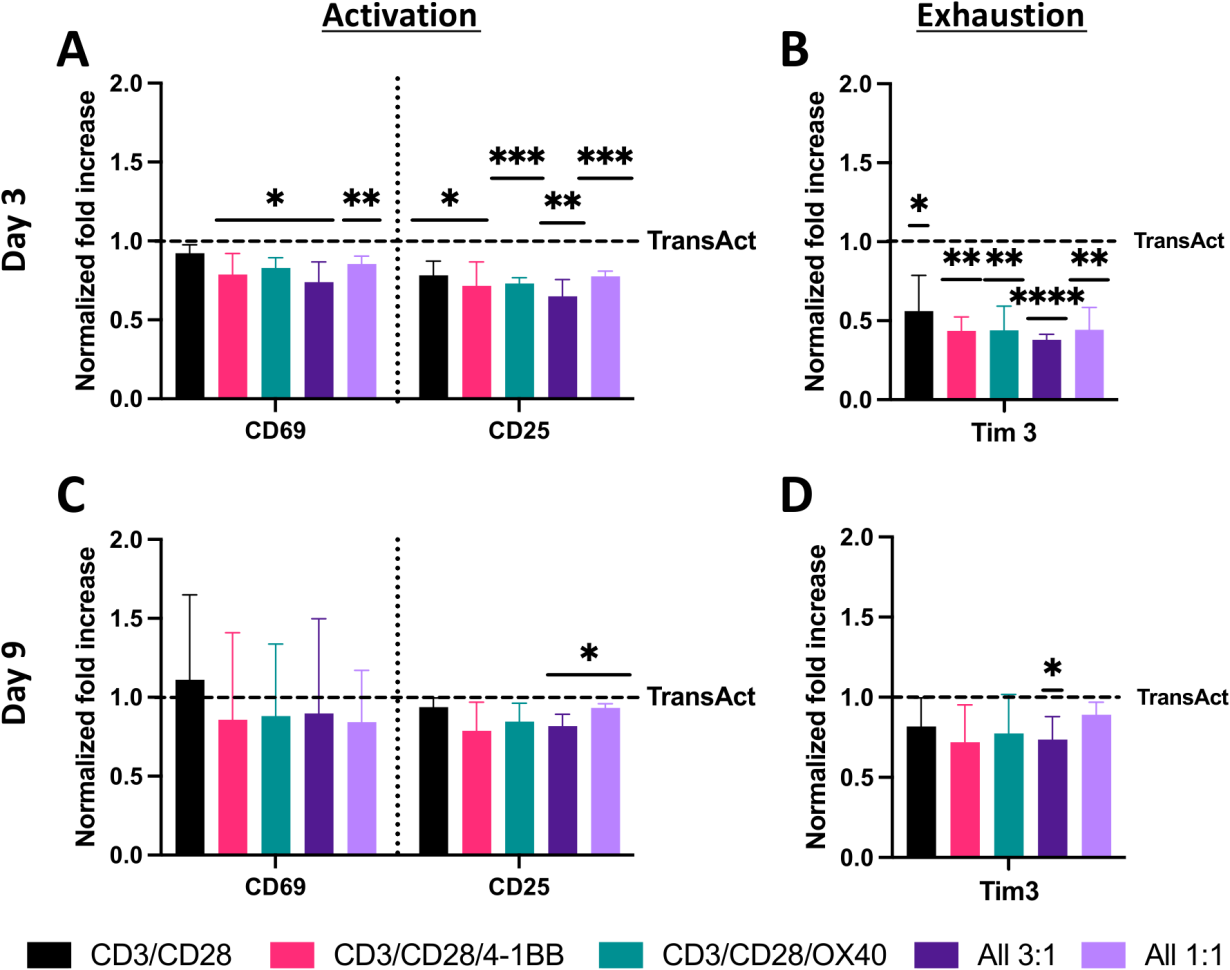
Analysis of T-cell activation and exhaustion using HCMs with different co-stimulatory molecules and TransAct as control. Data were normalized for each donor to TransAct for better understanding of significance and decrease of donor-to-donor variation. **(A)** Activation with CD69 and CD25 on day 3. **(B)** Exhaustion with Tim3 on day 3. **(C)** Activation with CD69 and CD25 on day 9. **(D)** Exhaustion with Tim3 on day 9. HCM color schemes: CD3/CD28 (black), CD3/CD28/4-1BB (pink), CD3/CD28/OX40 (green), All 3:1 (dark purple), All 1:1 (light purple). Four different donors with n≥3 for each donor were used as representative sample population. *p< 0.05, **p<0.01, ***p < 0.001 and ****p < 0.0001. Statistical significance was determined using one-sample Wilcoxon test to seek significant difference between the mean of HCMs and normalized TransAct value, and standard deviation is shown in all the graphs.

**Figure 5:**
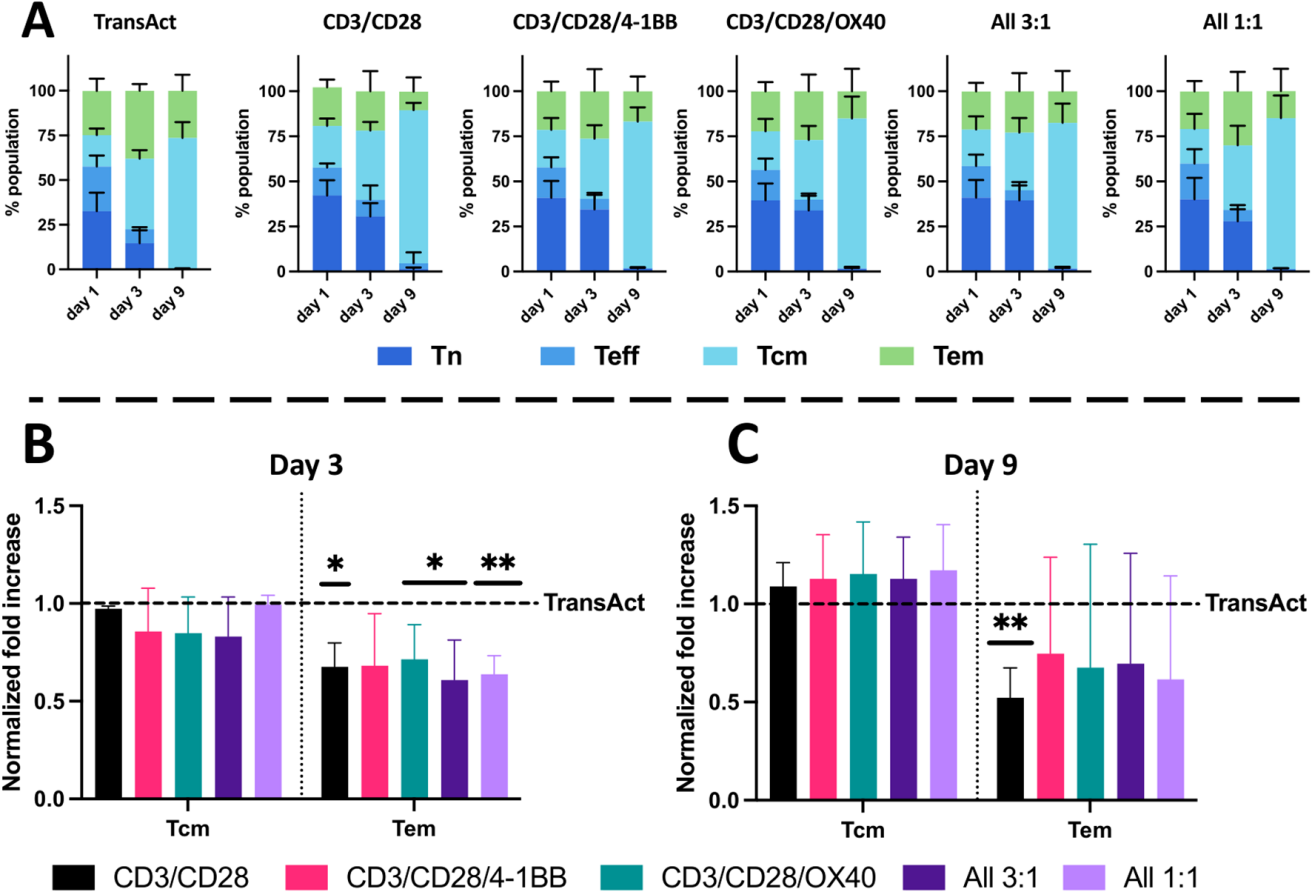
Analysis of T-cell phenotype using HCMs and TransAct. Phenotype analysis at days 1, 3, and 9 of the different populations for **(A)** TransAct, CD3/CD28, CD3/CD28/4-1BB, CD3/CD28/OX40, All 3:1, and All 1:1. Naïve population (Tn) represented in dark blue, effector population (Teff) in medium blue, central memory population (Tcm) in light blue, and effector memory population (Tem) in green. Phenotype after normalizing the data of each donor with TransAct on day 3 **(B)** and day 9 **(C)**. HCM color schemes: CD3/CD28 (black), CD3/CD28/4-1BB (pink), CD3/CD28/OX40 (green), All 3:1 (dark purple), and All 1:1 (light purple). Four different donors with n≥3 for each donor were used as representative sample population. *p< 0.05, **p<0.01. Statistical significance was determined using one-sample Wilcoxon test to seek significant difference between the mean of HCMs and normalized TransAct value, and standard deviation is shown in all the graphs.

**Figure 6:**
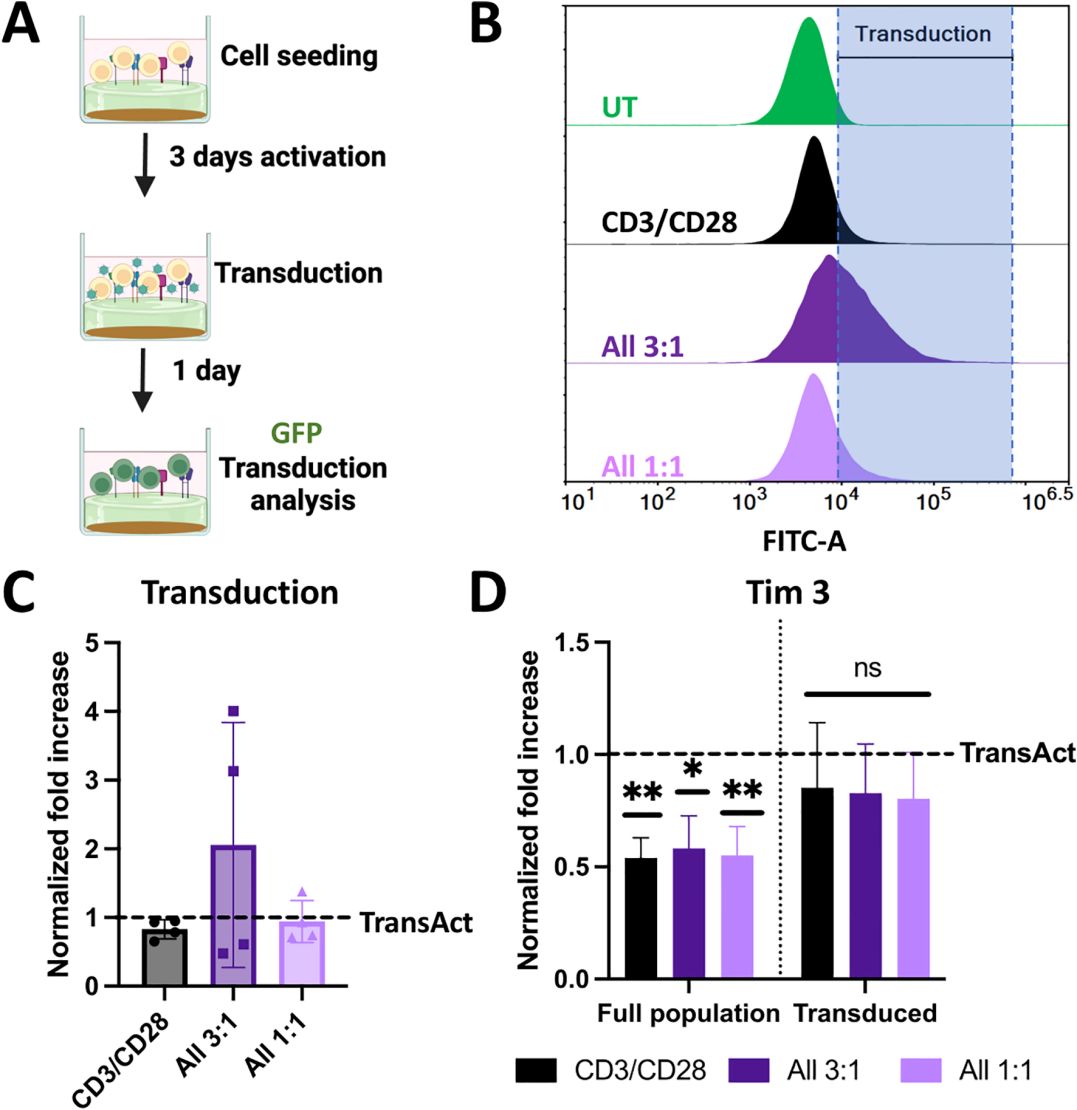
**(A)** Schematic representation of transduction experiments using a model GFP lentivirus. Created with Biorender. **(B)** Representative histograms of GFP fluorescence intensity for cells transduced with different HCMs conditions compared to untreated sample (with gating at <1% population). Increase in the fluorescence intensity corresponds to transduction, represented by the shaded area. **(C)** Normalized transduction to TransAct after 24 h. **(D)** Normalized Tim3 exhaustion to TransAct after 24 h for the full population and transduced population. Color schemes: CD3/CD28 (black), All 3:1 (dark purple), and All 1:1 (light purple). Four different donors with n≥3 for each donor were used as representative sample population. *p< 0.05, **p<0.01. Statistical significance was determined using one-sample Wilcoxon test to seek significant difference between the mean of HCMs and normalized TransAct value, and standard deviation is shown in all the graphs.

**Figure 7:**
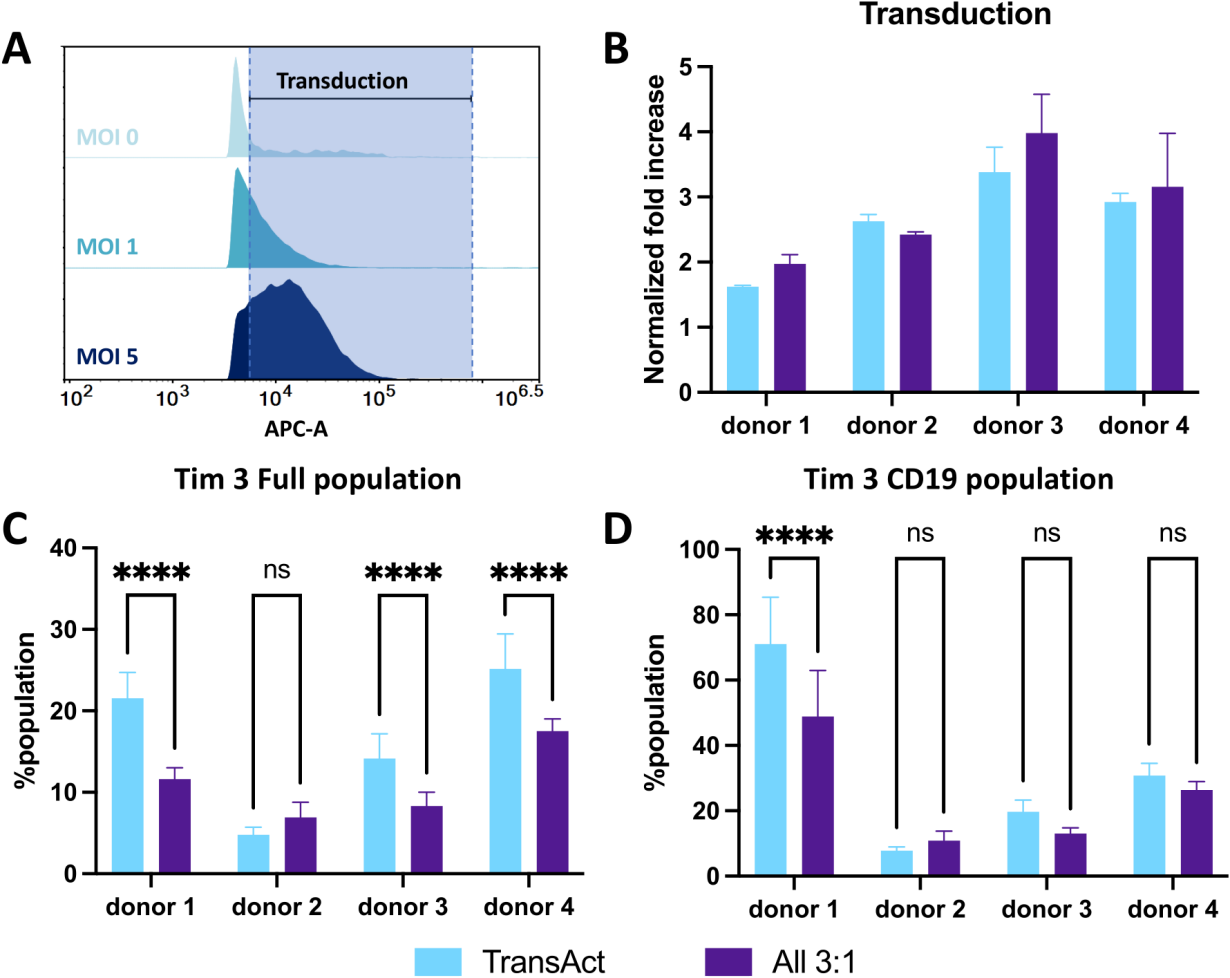
**(A)** Representative histograms of APC fluorescence intensity for cells transduced with different MOI conditions compared to MOI 0 sample using a <1% population as the gating strategy, with a CD19 lentivirus. Increase in the fluorescence intensity corresponds to transduction, represented by the shade area. **(B)** Normalized fold increase to MOI of 1 in transduction between TransAct (light blue) and All 3:1 HCM (dark purple) for each donor. **(C)** Tim3 exhaustion for the full population compared between TransAct and All 3:1 HCM. **(D)** Tim3 exhaustion for the transduced population compared between TransAct and All 3:1 HCM. Four different donors with n≥3 for each donor were used as representative sample population. ****p < 0.0001. Statistical significance was determined using two ways ANOVA with post-Tukey test, and standard deviation is shown in all the graphs.

In each experiment described in the Methods (Sections 2.5-2.9), four different donors were used, with four technical replicates per donor for each condition. Data normalization was performed to allow statistical comparisons and account for variability between donors and experiments. For experiments involving primary T cell activation (**Figures 2**, **4**, **5**), results for each donor were normalized to the average TransAct value for that donor: this normalization was done by dividing the individual technical replicate value by the average TransAct control value for each HCM condition. For experiments involving transduction with a model lentivirus (**Figure 6**), results for each donor similarly were normalized to the average TransAct control value for that donor. For experiments involving transduction with a CD19 CAR lentivirus (**Figure 7**), the MOI of 1 served as the control during normalization: for each of the donors, all TransAct data were normalized to the averaged MOI 1 of the TransAct condition, and All 3:1 data were normalized to the averaged MOI 1 of the All 3:1 condition. Before normalization, an outlier test (Robust Regression and Outlier Removal (ROUT) method in GraphPad Prism) was performed to control for potential error within samples. No values were identified as outliers.

## 3 Results

### 3.1 Activation of primary T cells

Well-defined hydrogel biomaterials offer the opportunity to mimic the biophysical and biochemical properties of the native microenvironment where T cells are activated (37). Specifically, here, hydrogels were formed by photopolymerization of PEGdiPDA on membranes to create substrates with physiologically-relevant moduli and that integrate an acryl-PEG-biotin group for the modular addition of desired bio-functionality to the system using biotin-avidin chemistry (**Supplementary Figures S1**-**S3**) (37). Different combinations of co-stimulatory molecules were investigated with a constant concentration of 20 μg·mL^-1^ total antibody combination for easy comparison between the different designed groups (**Figure 1B**), where 20 μg·mL^-1^ of 1:1 anti-CD3:anti-CD28 had previously been shown generally effective for T cell activation (30, 32). The importance of four different receptor binding ligands then was studied using anti-CD3 and anti-CD28 in combination with anti-4-1BB and anti-OX40 at varied ratios (**Figure 1B**): 10 μg·mL^-1^ CD3 with equal balance of other costimulatory ligand(s) for 20 μg·mL^-1^ total in *i)* CD3/CD28, *ii)* CD3/CD28/4-1BB, *iii)* CD3/CD28/OX40, *iv)* CD3/CD28/41BB/OX40 (All 3:1) and *v)* 5 μg·mL^-1^ each for all co-stimulatory ligands (All 1:1). TransAct, which is functionalized with anti-CD3/CD28, was used as a control as an industry standard for T cell activation. The capabilities of functionalized HCMs with different co-stimulatory molecules were studied with isolated primary human CD3+ T cells from four donors (**Supplementary Figure S7**) (38, 39).

When designing biomaterials for CAR T production, activation and expansion are critical metrics to characterize the potential to achieve a clinical grade of CAR T-cells (14). Therefore, proliferation, cell counts, and viability were examined at day 1, 3, and 9 during the experiment (**Figures 1C**, **2A**). T cell proliferation can be used as a secondary indicator for T cell activation, since cells that are not activated will have limited proliferation (40). Hence, proliferation index (PI) was used to quantify the number of divisions over time compared to a day 1 undivided sample (**Supplementary Figure S8**) (40). For all the samples, the PI was significantly higher on day 9 compared to day 3, indicating successful activation and increase of proliferative phenotypes (**Figure 2B**). On day 3, there were no observed significant differences between any of the HCM conditions and TransAct (**Figure 2C**); however, three of the HCM conditions showed lower proliferation compared to TransAct on day 9 (**Figure 2D**). A similar trend can be observed in the total number of cells on day 3 and day 9, where CD3/CD28/4-1BB and CD3/CD28/OX40 had statistically lower cell counts than TransAct or CD3/CD28 (**Figure 2E**). However, when normalizing cell counts to TransAct for each donor, no significant differences were found (**Figure 2F**). Additionally, viability of our T cells was higher than 85% with all the combinations, showing no statistical differences with TransAct (**Supplementary Figure S10**).

The conditions that had the lowest proliferative profiles by day 9 were CD3/CD28/4-1BB, CD3/CD28/OX40, and All 3:1. Further, lower cell counts were observed for CD3/CD28/4-1BB and CD3/CD28/OX40 but not the All 3:1 combination (**Figure 2E**). Based on these observations, we hypothesize that combining anti-41BB and anti-OX40 is needed for co-stimulation and robust activation, as they do not have a strong enough proliferative and activating profile to be used only in combination with anti-CD3 and anti-CD28 co-stimulatory molecules. Taking a deeper look at the All 3:1 HCM combination, one can compare the two HCM compositions that have all of the co-stimulatory molecules but in different ratios: All 1:1 has equal amounts of all 4 costimulatory ligands, whereas All 3:1 has equal amounts of anti-CD28/4-1BB/OX40 and a higher amount of anti-CD3. When comparing both All combinations with our TransAct mimic (CD3/CD28), the importance of including anti-4-1BB and anti-OX40 and differential amounts of anti-CD3 and anti-CD28 becomes evident, demonstrating the value of the modular HCM platform and the potential synergistic effect of using all the molecules together to achieve successful activation.

Given our initial findings that combinations of HCMs could yield robust expansion of T cells, we next investigated the resultant cellular phenotypes using flow cytometry at day 1, 3, and 9 (**Figures 1C**, **3**, **Supplementary Figures S11**-**S15**). Early activation was studied using the type II C-lectin receptor CD69, where an initial increase on day 1 and day 3 was observed with a subsequent decrease by day 9 (**Figure 3** **left**). Accordingly, late activation, measured by the α-subunit of the IL-2 receptor CD25, exhibited an opposite trend, with low CD25 expression on day 1 but high expression on days 3 and 9. Phenotypical analysis indicated a general trend in increasing memory phenotypes over time, as expected (30). Exhaustion was analyzed by measuring T cell immunoglobulin and mucin domain-containing protein 3 (Tim3) expression, finding a general increase over time (**Figure 3** **center**). Phenotype was characterized using the combination of L-selectin (CD62L) and CD45RO expression (17), where we define Tn as CD62L^hi^ CD45RO^lo^, Teff as CD62L^low^ CD45RO^lo^, Tcm as CD62L^hi^ CD45RO^hi^, and Tem as CD62L^lo^ CD45RO^hi^ (**Figure 3** **right**). Data were normalized to TransAct to remove donor-to-donor variability and enable comparison between each combination of co-stimulatory molecules on the HCMs (**Figure 4**), where the complete population analysis can be found in **Supplementary Figure S16** for day 3 and **Supplementary Figure S17** for day 9.

T cells on all HCM compositions exhibited a statistical decrease in early- and late-stage activation compared to TransAct on day 3 (**Figure 4A**). This lower activation rate was correlated with lower exhaustion on day 3 for T cells that were activated using HCMs (**Figure 4B**). Notably, the combination of CD3/CD28, the same co-stimulatory molecules as TransAct, had the highest activation as well as the highest exhaustion of the HCM combinations on day 3. Day 3 is the transduction step in the general CAR T manufacturing process, making this time point of particular interest to minimize exhaustion (30, 41). By day 9, all HCM conditions had a similar early- and late-stage activation to TransAct (**Figure 4C**), with the exception of the All 3:1 and All 1:1 conditions, which showed slightly lower late-stage activation. Importantly, the lower exhaustion observed on day 3 translated into the All 3:1 condition having statistically lower exhaustion on day 9 (**Figure 4D**). This observation was noteworthy since the All 3:1 combination maintained the concentration of anti-CD3 at 10 μg·mL^-1^ and decreased the concentration of anti-CD28 to 3.33 μg·mL^-1^ in addition to incorporating anti-4-1BB and anti-OX40 at the same concentration of 3.33 μg·mL^-1^. In these data, the addition of anti-CD3 and anti-CD28 for achieving a strong late-stage activation is evident, as already noted on PI and cell count experiments; however, this strong activation may also contribute to exhaustion. The use of other co-stimulatory molecules with lower induction of activation (e.g., anti-4-1BB and anti-OX40), as well as decreased concentrations of anti-CD28, provides sufficient activation (e.g., proliferation, phenotype) while decreasing exhaustion in early and late time points.

The next step was to analyze population phenotypical changes of T cells during activation with HCMs. To characterize the shifts in the full population from naïve and effector phenotypes to memory phenotypes, the populations on day 1, 3, and 9 were represented next to each other for each HCM condition and TransAct. As can be observed in **Figure 5A**, T-cell phenotype consistently shifted from a predominantly naïve phenotype to memory phenotypes (Tcm and Tem). When comparing with TransAct on day 9, we also observe that HCM conditions generally show a trend of a greater fraction of Tcm and smaller fraction of Tem. After observing this general trend, we normalized the fraction of memory phenotypes to TransAct at day 3 and day 9 to normalize the donor-to-donor variability (**Figures 5B, C**). On day 3, a significant decrease of Tem cells can be observed compared with TransAct with a similar Tcm phenotype; this was due to the general larger number of naïve T cells remaining in the HCMs. The increase of Tn at early time points (day 1 and day 3) can also be associated with the slower but more uniform activation achieved by HCMs as shown in **Figure 4**. When comparing the memory phenotypes on day 9, all of the HCM conditions show a trend of increased Tcm (as indicated by averaged normalization values greater than one) and decreased Tem cells (as indicated by averaged normalization values less than one) (**Figure 5C**). With the exception of the CD3/CD28 HCM, these trends were not statistically significant, owing in part to donor-to-donor variability. However, in aggregate, our results support that HCMs promote a gradual and uniform shift from naïve T cells to a predominant central memory phenotype by day 9, where a central memory phenotype is desirable for CAR T efficacy (21, 22).

### 3.2 Transduction of primary T cells

In our activation experiments, we observed that T cells activated with HCM combinations of CD3/CD28, All 3:1, and All 1:1 led to significantly lower T-cell exhaustion and Tem phenotype on day 3; hence, we decided to focus on those combinations moving forward and not pursue further experiments with CD3/CD28/OX40 and CD3/CD28/4-1BB conditions. We next aimed to understand how these differences in activation impact viral transduction. For transduction experiments, cells were activated for 3 days with HCMs or TransAct; after 3 days, transduction was performed using a model GFP lentivirus with an MOI of 1, and GFP production was characterized using flow cytometry after 24 hours (**Figure 6A**). Transduction percentage was established using an untreated sample (not transduced) to gate at a <1% positive population as shown in the representative histograms (**Figure 6B**). For ease of comparison and due to donor-to-donor variability, GFP+ expression was normalized to TransAct (data without normalization can be found in **Supplementary Figure S18**), showing each donor with a dot on the bar graph (**Figure 6C**). The combination of CD3/CD28 had a similar transduction profile as TransAct, as expected since both systems use the same co-stimulatory molecules. Interestingly, while some donors in the combinations of All 3:1 and All 1:1 had similar transduction to TransAct, a few conditions showed notable improvements. In particular, the All 3:1 HCM had two donors with a 3-to-4-fold increase in transduction. This observation may be attributable to the increase in naïve T cells on the All combinations compared to TransAct (**Figures 5A, E, F**), where an increase in naïve populations has been linked to stem-like memory phenotypes increasing transduction (22, 42). Taking a deeper look at the exhaustion profiles of the non-transduced and transduced populations, cells activated with HCMs show a decrease in Tim3 expression that is also reflected in the transduced population (**Figure 6D**).

Given the promising transduction of the All 3:1 combination compared to the other two combinations tested, we sought to translate these results to a clinically relevant virus. A third-generation CD19-CAR lentivirus (Vector BioMed) was used to generate CD19-CAR T-cells. Transduction experiments were performed following the same workflow used with the GFP lentivirus (**Figure 6A**) and two different MOI conditions were tested (MOI 1 and 5). Transduction percentage was based on gating the untreated sample (not transduced) at <1% positive population (**Figure 7A**), and an increase in CD19-CAR transduction was successfully achieved between MOI 1 and MOI 5. To avoid discrepancies in CD19-CAR expression due to batch-to-batch variability, CD19+ expression was normalized to the MOI 1 condition for each donor (data without normalization can be found in **Supplementary Figure S19**). An MOI of 5 was used to compare CD19+ expression for each donor between the All 3:1 HCM and TransAct activation. Three of the four donors demonstrated improved transduction when using the All 3:1 HCM combination (**Figure 7B**), confirming the potential of HCM-driven activation to increase transduction. Tim3 was again used to characterize exhaustion of the full population (**Figure 7C**) and the transduced population (**Figure 7D**). Three out of four donors showed a significant decrease in Tim3 expression in the full population, with one donor showing significantly reduced exhaustion in the transduced CD19 population. While not significant for all the donors after transduction, a general decrease in exhaustion with the All 3:1 HCM condition relative to Transact could be observed for the majority of donors. Note that the transduced CD19 population for each donor generally shows an increase in the percentage of the population exhibiting Tim3 after transduction. This finding is consistent with literature that shows a general increase in exhaustion of CAR T-cells after transduction (43, 44), reiterating the importance of approaches to promote lower exhaustion profiles during activation for CAR T production.

### 3.3 Antigen specificity of CAR T-cells produced with HCMs

**Figure 8:**
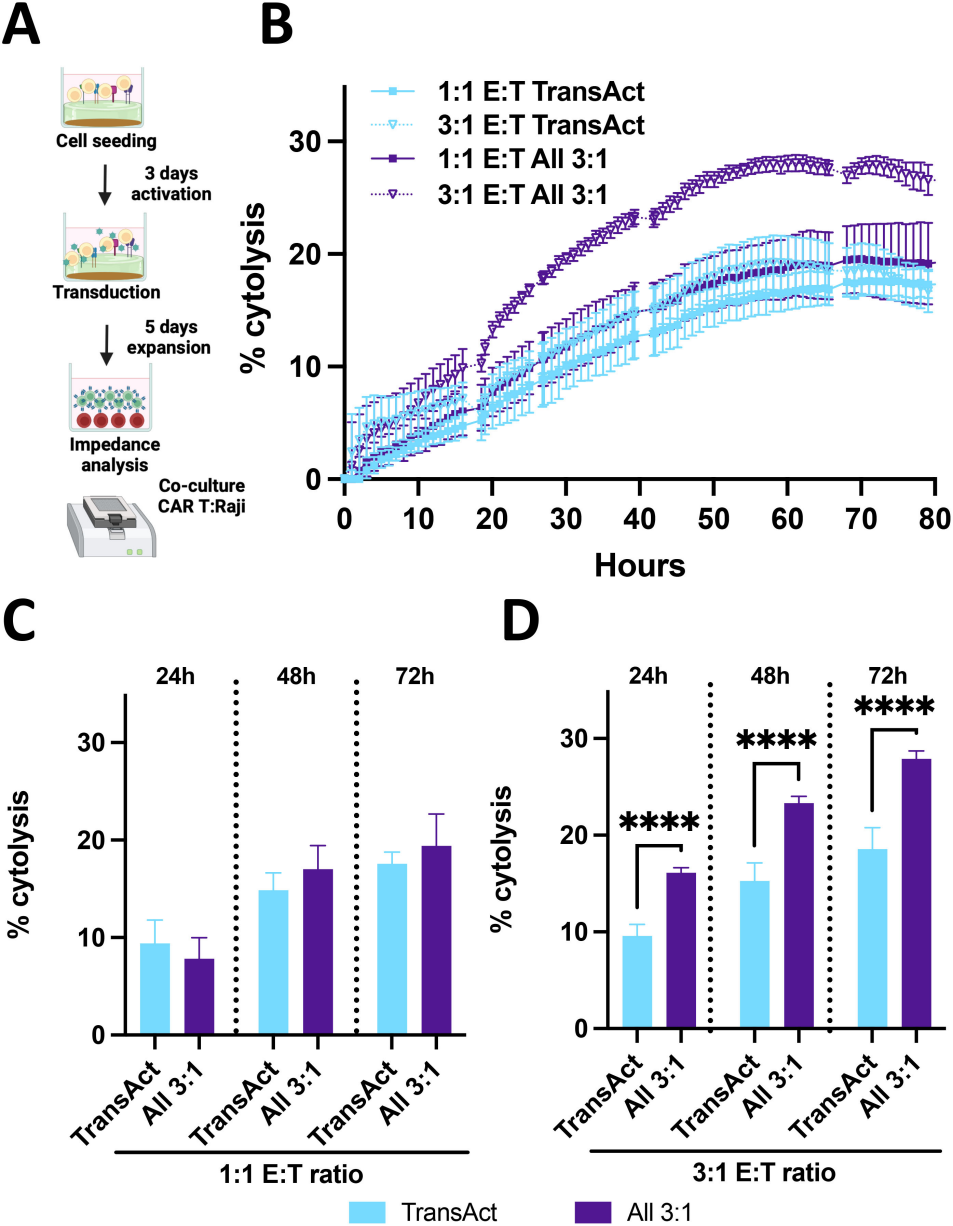
**(A)** Schematic representation of killing assay using impedance measurements. Created with Biorender. **(B)** % cytolysis over time with measurements at every hour for 70 hours where cytolysis increase can be observed. TransAct represented with light blue and All 3:1 represented with dark purple. Different E:T ratios were studied, 1:1 E:T represented with a square and 3:1 E:T ratio represented with a triangle and dotted line. **(C)** % cytolysis at 24, 48, and 72 h comparing All 3:1 and TransAct activation of CAR T-cells after transduction with CD19 virus with 1:1 E:T ratio. **(D)** % cytolysis at 24, 48, and 72 h comparing All 3:1 and TransAct activation of CAR T-cells after transduction with CD19 virus with 3:1 E:T ratio. One donor with n≥4 was used as representative killing potential. Statistical significance was determined using T-test, and standard deviation is shown in all the graphs, ****p < 0.0001.

The final step in assessing the function of the CAR T-cell product was to compare the *in vitro* killing potential between CAR T-cells activated with the industry standard (TransAct) and the All 3:1 HCM condition. Raji-Luc2 cells (a CD19+ leukemia model cell line) were used as target cells and were co-cultured with CAR T-cells produced by activation with TransAct or All 3:1 HCM condition. To determine the viability of Raji cells during co-culture, impedance measurements were used (45), measured with xCELLigence (Agilent) and analyzing Raji-Luc2 cells attached to the bottom of the plate (**Figure 8A**). These data were transformed to % cytolysis by using a healthy Raji-Luc2 control and subtracting the decrease in impedance when using CAR T-cells (**Figure 8A**). For this experiment, T cells were activated with TransAct or All 3:1 HCMs conditions and transduced after 3 days of activation with an MOI of 5. We achieved an 18% transduction for All 3:1 and 13% for TransAct conditions, as measured at day 4; after transduction, cells were expanded until day 9 and then killing efficacy was assessed. Two different Effector to Target (E:T) ratios were studied, 1:1 and 3:1 E:T, defined as 1 CAR T-cell per 1 Raji cell and 3 CAR T-cells per 1 Raji cell. As expected, there was an increase in killing potential by increasing the E:T (i.e., increasing the number of CAR T-cells for both conditions tested) (**Figure 8B**).

To further compare the differences between TransAct and All 3:1, the two different E:T ratios were plotted separately, and three relevant time points from the continuous experiment were selected. For the 1:1 E:T ratio (**Figure 8C**), we observe that CAR T-cells from TransAct and the All 3:1 HCM have a similar effect against Raji cells. Notably, when comparing the killing potential at a higher E:T ratio, the benefits of using the All 3:1 HCM combination for T-cell activation become evident, achieving a statistically significant increase in cytolysis for all the time points studied compared to TransAct. When looking at the final timepoint (72 h), All 3:1 achieved a 28% cytolysis with small variability, while TransAct achieved a 19% cytolysis with larger deviation (**Figure 8D**). These results were also corroborated by image analysis using confocal microscopy and counting the number of Raji cells previously labeled with cell trace (**Supplementary Figure S9**), with a 24% cytolysis using All 3:1 and 11% cytolysis using TransAct. After demonstrating the efficiency of CAR T-cells activated with the well-defined HCMs with the All 3:1 combination, we tested their potential with a different donor at the 3:1 E:T ratio, achieving a consistent increase in killing potential with lower standard error compared to TransAct (**Supplementary Figure S20**).

## 4 Discussion and conclusions

In this work, we report the use of well-defined bio-inspired hydrogels as a tunable platform to present arrays of co-stimulatory molecules that can achieve modular control over resultant T-cell activation, exhaustion, phenotype, transduction, and killing efficacy. We find that permutations of anti-CD3, anti-CD28, anti-4-1BB, and anti-OX40 conjugated on the biotin pendant groups of the HCM result in controlled, uniform activation, a preference towards the Tcm phenotype, and limited exhaustion of T cells, which together support production of CAR T-cells with equivalent to superior performance when compared to the industry standard TransAct control. Our work lays a critical foundation for the design of HCMs for a CAR T manufacturing application, as the HCMs are readily integrated within a scalable TFF flow device demonstrated to improve T-cell transduction in addition to scalability of the HCMs themselves (30). Moreover, this work lends insight into critical requirements for synthetic T-cell activation while highlighting the modularity and tunability of our HCM approach.

Our choices of co-stimulatory ligands were inspired by APC-mimicry. The CD3 T-cell receptor (TCR) complex is the first signal that T cells use natively to initiate activation (16). However, for successful T-cell activation, TCR engagement is not enough, and co-stimulatory signaling is necessary (31). CD28 is commonly provided in conjunction with CD3 as it serves as an amplifier of TCR signaling; it is expressed in naïve T cells and has been linked to an increase in cytokine production promoting T-cell survival (31, 32). 4-1BB is a membrane protein expressed in T cells and has been shown to enhance T-cell signaling related to survival by suppressing activation-induced cell death by increasing antiapoptotic gene expression (e.g., bcl-x(L), c-FLIP, and bfl-1) (33). Therefore, the use of 4-1BB was hypothesized to have the potential to decrease the expression of exhaustion markers during the CAR T manufacturing process, which is supported by our results shown in **Figures 4** and **6**. Furthermore, CD28 and 4-1BB are known to work synergistically to promote cytokine production including IL-2, INF-γ, and TNF-α that promote proliferation of CD8 T-cells (33, 46–48). Lastly, OX40 was selected as it has been associated with activated CD4+ and CD8+ T-cells (34) and has the potential to enhance anti-tumor immunity by promoting T cell infiltration, a general limitation of CAR T therapies (49, 50). OX40 is a transmembrane protein that has been shown to be fundamental for T-cell activation in later stages of immune response, playing a critical role to enhance function of effector T-cells and increasing survival (34). Overall, our data support superior performance of the All 3:1 combination, highlighting the benefit of all four of these molecules towards creating an optimized T-cell product (34). Interestingly, inclusion of either anti-4-1BB or anti-OX40 with the anti-CD3 and anti-CD28 on HCMs yielded lower proliferation than combinations with both anti-4-1BB and anti-OX40 (19). We hypothesize that anti-41BB and anti-OX40 alone do not have a strong enough proliferative and activation profile, but rather behave synergistically, suggesting that anti-41BB and anti-OX40 need to be combined to achieve sufficient activation. Moreover, our results also support the importance of anti-CD28 for robust activation, while being a driver of T-cell exhaustion.

Throughout these studies, activation on the HCMs relative to TransAct yielded T cells with *i)* lower exhaustion in all samples tested (**Figure 4**) with statistical differences for all compositions at day 3 and for All 3:1 composition at day 9; *ii)* improved phenotype (i.e., decreasing Tem) (**Figure 5**) with statistical differences for most compositions at day 3 and for CD3/CD28 at day 9; *iii)* similar or enhanced transduction (**Figures 6**, **7**) with a trend of increased transduction for All 3:1; and *iv)* relevant function with statistical increases in killing efficacy over time for All 3:1 (**Figure 8**). Transduction with a relevant virus is a fundamental step of the CAR T manufacturing process (51); therefore, a model GFP lentivirus was used to demonstrate the potential of HCM-activated T-cells, achieving a 3-4-fold increase for the All 3:1 combination for two of the donors tested (**Figure 6**). This increase in transduction was associated with the decrease in Tim3 expression for our HCMs combinations. The presence of Tim3 is an indicator of T-cell exhaustion: when T cells become exhausted, they lose function, proliferative capacity, and effector function (52, 53). This loss of function and proliferative capacity was associated with the lower transduction potential of TransAct-activated T-cells. Moreover, lentiviral vectors need cell division to infect T-cells; hence, it is critical for proliferation and cells in G(1b) phase of the cell cycle for successful transduction (54). Reduced exhaustion thus is not only important for CAR T survival in the body, as noted in the literature, but also in T-cell transduction. Notably, the lower exhaustion observed in T-cells activated on HCM was also comparable in the transduced population (CAR T population) (**Figure 7**). The significantly lower exhaustion, the high fraction of memory phenotypes (all Tem or Tcm), and the improved ratio of Tcm to Tem populations that were produced from the All 3:1 HCM combination translated to a more efficacious CAR T-cell product for killing of target cells (**Figure 8**). Additionally, consistency of CAR T-cells produced by HCMs proved to be higher than TransAct activated CAR T-cells, for two different donors. Overall, these results speak to the importance of a controlled, homogeneous activation of T cells for a CAR T product with effective and consistent killing potential.

In summary, we have designed a bio-inspired modular platform based on scalable, well-defined hydrogels with physiologically-relevant moduli for the display of a range of co-stimulatory molecules to control T-cell activation, phenotype, and exhaustion. We have reported the importance of a controlled activation that is achieved by using differential amounts of anti-CD3 and anti-CD28 (most frequently used molecules for T-cell activation), decreasing the concentration of anti-CD28, and adding anti-4-1BB and anti-OX40 molecules. Decreased T-cell exhaustion is an advantage compared to current industry standards (e.g., TransAct) that translates into low exhaustion in the CAR T product. Additionally, the modest but significant increase *in vitro* killing efficacy observed shows the potential for applications of the produced, low-exhaustion CAR T-cells in individuals, with future opportunities for probing their use upon re-stimulation (29, 55). Furthermore, the hydrogel polymerization on top of a flat-sheet regenerated cellulose membrane allows for integration into our previously designed tangential flow filtration device, enabling the control provided by flow (e.g., virus concentration) as well as scalability of the manufacturing process (30). While the presented studies focused on the use of human cells and their killing efficacy *in vitro*, future experiments *in vivo* will be needed to determine the long-term success of the low-exhaustion CAR T products for translation. Note, all experiments were performed with cells from healthy donors, and significant differences have been reported in the literature between cells from healthy individuals and those with leukemia when producing CAR T-cells (e.g., lower cell counts, viability, expansion rates), presenting an opportunity for future investigations with this platform (56, 57). For further evaluation using cells from individuals with leukemia, immune status and genetic background also should be examined since these can have an effect on T-cell transduction, activation, and Tim3 expression (58, 59). The results achieved in this study are a first step to achieve low-exhaustion CAR T products with enhanced performance against cancer cells. The bio-inspired hydrogel platform has the potential to be introduced in the CAR T manufacturing process, exploiting its facile fabrication and modularity for controlled T-cell activation.

## Data Availability

The raw data supporting the conclusions of this article will be made available by the authors, without undue reservation.
