# Supplementary material for: Bio-functional hydrogel coated membranes to decrease T-cell exhaustion in manufacturing of CAR T-cells
Source: Front Immunol. 2025 Jun 27;16:1513148. doi: 10.3389/fimmu.2025.1513148 (PMC12245814; doi:10.3389/fimmu.2025.1513148)
Supplement: Supplementary file 1 [file DataSheet1.docx]

Supplementary Material

Bio-functional hydrogel coated membranes to decrease T-cell exhaustion in manufacturing of CAR T cells

Aida López Ruiz^1^, Eric D. Slaughter^1^, Kartik Bomb^1^, Samantha L. Swedzinski^2^, Paige J. LeValley^1^, Zaining Yun^1^, Jacob McCoskey^3^, Kara Levine^3^, Jonathan Steen^3^, Joseph Almasian^3^, Aparajita Chatterjee^3^, Christina Carbrello^3^, Dustin S. Chang^4^, Hubaida Fuseini^4^, Yama A. Abassi^4^, Abraham M. Lenhoff^1^, Catherine A. Fromen^1^,* and April M. Kloxin^1,2^,*

Departments of ^1^Chemical and Biomolecular Engineering and ^2^Materials Science and Engineering University of Delaware, Newark, DE; ^3^EMD Millipore Corporation, Bedford, MA, ^4^Agilent Technologies, Santa Clara, CA


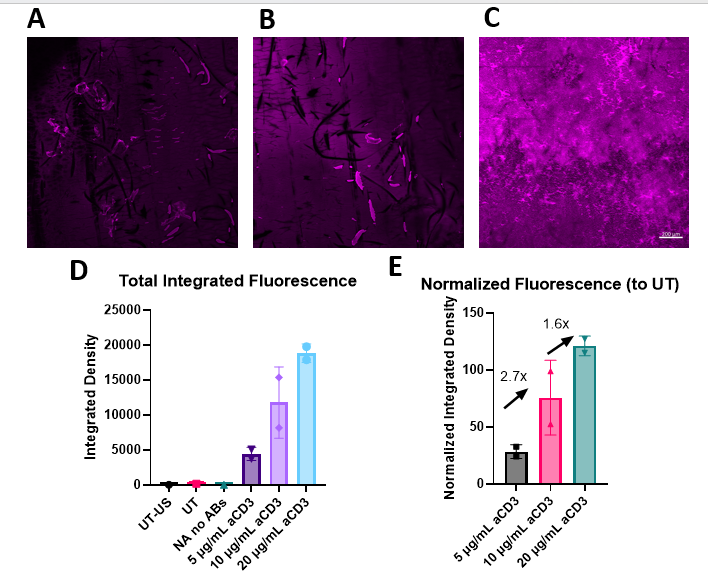


**Figure S1.** A-C). Confocal images of anti-CD3 HCMs stained with AF647 goat anti-mouse IgG1 at 5, 10, and 20 μg/mL of anti-CD3 (left to right respectively). D). Total integrated fluorescence of confocal images as determined in FIJI using the Plot Profile function of the 3 different anti-CD3 concentrations (5,10, and 20 μg/mL) and unstained HCM with untreated no anti-CD3 antibody/no neutravidin (UT-US), stained HCM with no anti-CD3 antibody/no neutravidin (UT), and stained HCM with no-anti-CD3 antibody and neutravidin (NA no ABs). E) 5, 10, and 20 μg/mL anti-CD3 HCM total integrated fluorescence, normalized to UT. Antibody activity, directly proportional to the integrated fluorescent density, shows an increase in fluorescence intensity with increased concentration. Error bars are standard deviation.

**Table S1.** Donor information.

| **Donor** | **Weight (lbs)** | **Age** | **Sex** | **Race** | **Smoker?** |
| --- | --- | --- | --- | --- | --- |
| A | 182 | 39 | M | B | N |
| B | 234 | 30 | M | B | N |
| C |  | 32 | M | B | N |
| D | 174 | 54 | M | B | N |
| E | 381 | 39 | M | B | N |
| F | 260 | 39 | M | B | N |
| G | 156 | 35 | F | B | N |
| H | 253 | 40 | F | B | N |

**Figure S2.** Representative ^1^H NMR Spectrum of LAP (Lithium Phenyl-2,4,6-trimethylbenzoylphosphinate), photoinitiator used for polymerization of HCMs (DMSO-d6, 400 MHz, 16 scans). δ 7.59 (m, 2H), 7.48 (m, 1H), 7.43 (m, 2H), 6.73 (s, 2H), 2.21 (s, 3H), 2.15 (s, 6H).

**Figure S3.** ^1^H NMR Spectra for PEGdiPDA from the three batches used during the presented work (DMSO-d6, 400 MHz, 128 scans). The protons associated with the photodegradable acrylate moiety (δ 7.91, 7.56, 7.14 ppm) were normalized to the PEG backbone protons (159H, δ 3.42-3.74 ppm), and the functionality was then estimated to be 83% (top spectrum), 97% (middle spectrum), or 94% (bottom spectrum). Specifically, functionality (F) was calculated by dividing the integration (X*_n_*) of these three peaks by their ideal theoretical value (Y*_n_*) (*n=1,* 2, or 3), taking the average, and then multiplying by 100% to obtain the percent functionality: for example, to calculate the functionality for the first batch of PEGdiPDA, F = [(0.80/1)+(0.86/1)+(0.84/1)]/3 *100% =83%. δ 7.91 (s, 1H), 7.55 (s, 1H), 7.14 (s, 1H), 6.37 (s, 1H), 6.30 (s, 1H), 6.22 (s, 1H),5.98 (s, 1H), 4.05 (s, 2H), 3.90 (s, 3H), 3.50 (s, 159H), 3.40 (s, 3H), 3.20 (s, 2H), 2.24 (s, 2H), 1.61 (s, 3H).


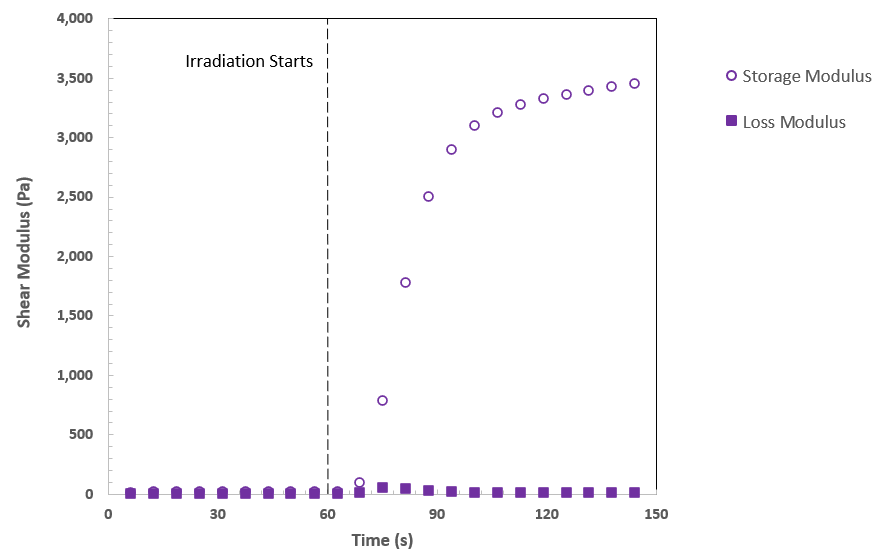


**Figure S4.** Storage (G’) and Loss (G’’) modulus of hydrogels formed in situ on a rheometer, as determined by oscillatory rheology. Average of three replicates of monomer solution are shown here, where irradiation (5 mW cm^-2^ at 400-500 nm) was started at 60 seconds. Elastic modulus can be estimated using rubber elasticity theory (E ~ 3*G) for these hydrogels, demonstrating an in situ Young’s modulus on the order of ~10 kPa.

**Figure S5.** A) XZ plane images of HCMs for viewing the height of hydrogel coatings over time with confocal microscopy. Representative images of hydrogel coatings measured over time for height tracking with incubation at 4 ºC and 37 ºC (physiologically relevant condition). No changes were observed visually in the appearance of the hydrogel coating. B) Quantification of the height of hydrogel coatings over time (incubation at 4 ºC (black) and 37 ºC (pink)) using confocal microscopy and Image J. The hydrogel height is consistent throughout the experimental time course, indicating stability of HCMs over time including at physiologically relevant conditions. Data points represent the mean and error bars represent standard deviation.


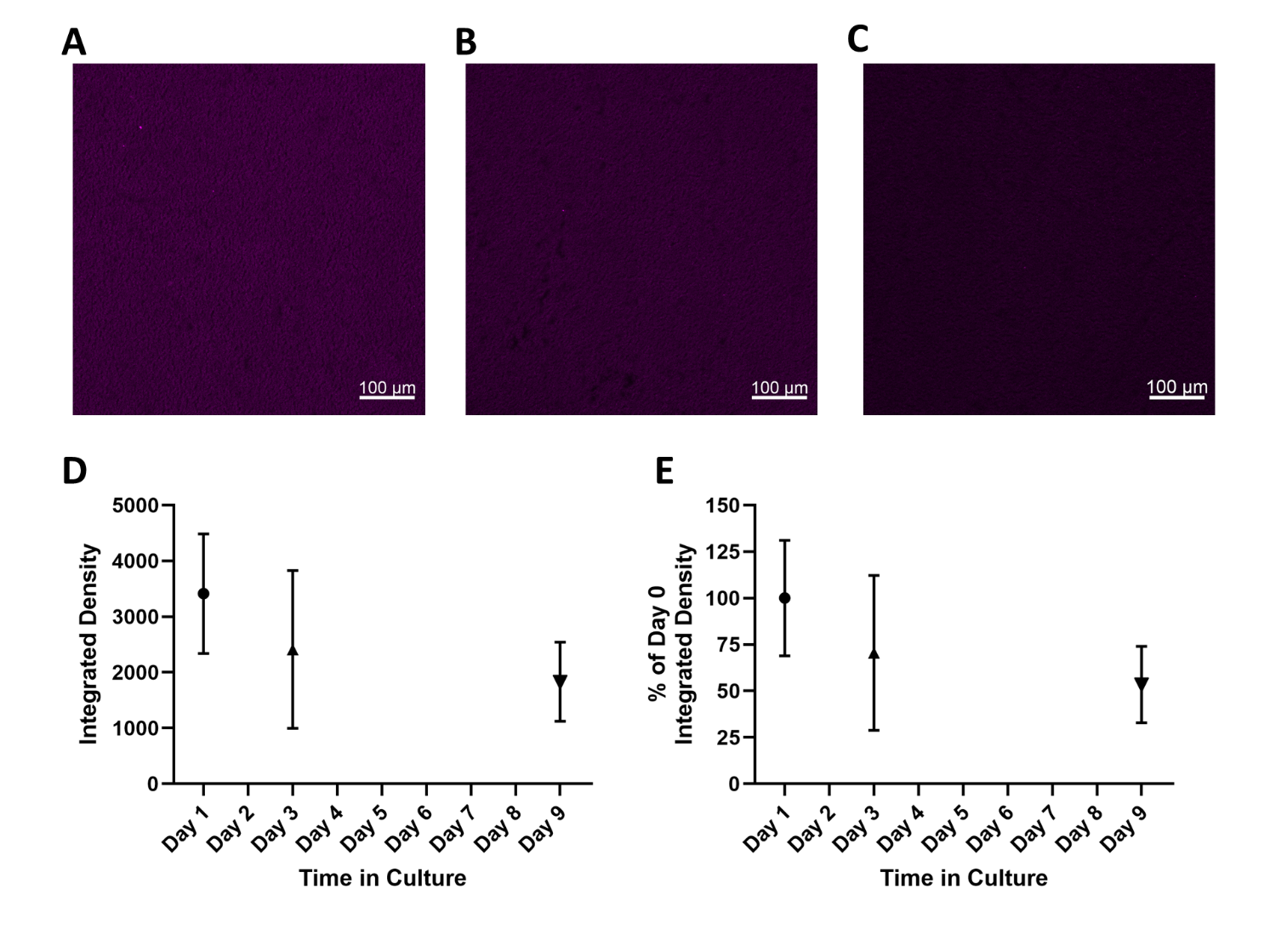
**Figure S6.** A-C) Representative confocal images of HCMs functionalized with anti-CD3 (20 μg·mL^-1^); incubated at 37 ºC with 5% CO2 in complete culture medium for 1 (A), 3 (B), and 9 (C) days, respectively; and then immunostained and imaged with confocal microscopy. D) Quantification of total integrated fluorescence of confocal images using FIJI. E) Total integrated fluorescence normalized to the average day 1 integrated fluorescence. Antibody concentration, directly proportional to the integrated fluorescent density, is consistent over the experimental time course within measurement error, supporting stability of the antibody functionality on the HCM. Data points represent the mean and error bars represent standard deviation.

**Figure S7.** (Top) Flow cytometry gating scheme for CD3+ isolation. Flow chart shows all events and gated cells on the left; from the cells gate, the singlets were gated. Based on singlets, the dead cells were gated out using the Zombie Red live/dead stain. (Bottom) Two samples before (left) and after (right) CD3 isolation indicate a 97% CD3+ isolation.

**Figure S8.** Representative histograms of proliferation index (PI) for day 1, 3, and 9 with All 3:1 HCM combination. Pink line is used to determine the initial population before cellular divisions on day 1, and blue line is used to mark the lack of proliferation dye on cells, which will mean that all the cells have divided under the detection limit of the instrument.

**Figure S9.** Representative images of stained Raji cells with cell trace after 72h co-culture with CAR T cells. Untreated control is a monoculture of Raji cells, and positive control is a monoculture of Raji cells with a lysis agent used to ensure cell death. All 3:1 and TransAct conditions were studied at two different effector-to-target ratios, 1:1 and 3:1.

**Figure S10**. Viability of T-cells after activation for 1, 3, and 9 days with TransAct or HCMs assessed using Trypan Blue. Three different donors with n≥3 for each donor were used as representative sample population, where standard deviation is shown in the graph.

**Figure S11.** Representative flow cytometry data for phenotype, activation (early and late), and exhaustion achieved using HCMs functionalized with CD3/CD28, with the same donor. Flow cytometry profiles are representative of sample changes for the same donor through the duration of activation experiments for days 1, 3, and 9. Orange arrows highlight the increase or decrease of the studied markers through the total length of the experiments.

**Figure S12.** Representative flow cytometry data for phenotype, activation (early and late), and exhaustion achieved using HCMs functionalized with CD3/CD28/4-1BB, with the same donor Flow cytometry profiles are representative of sample changes for the same donor through the duration of activation experiments for days 1, 3, and 9. Orange arrows highlight the increase or decrease of the studied markers through the total length of the experiments.

**Figure S13.** Representative flow cytometry data for phenotype, activation (early and late), and exhaustion achieved using HCMs functionalized with CD3/CD28/OX40, with the same donor. Flow cytometry profiles are representative of sample changes for the same donor through the duration of activation experiments for days 1, 3, and 9. Orange arrows highlight the increase or decrease of the studied markers through the total length of the experiments.

**Figure S14.** Representative flow cytometry data for phenotype, activation (early and late), and exhaustion achieved using HCMs functionalized with All 3:1, with the same donor. Flow cytometry profiles are representative of sample changes for the same donor through the duration of activation experiments for days 1, 3, and 9. Orange arrows highlight the increase or decrease of the studied markers through the total length of the experiments.

**Figure S15.** Representative flow cytometry data for phenotype, activation (early and late), and exhaustion achieved using HCMs functionalized with All 1:1 with the same donor. Flow cytometry profiles are representative of sample changes for the same donor through the duration of activation experiments for days 1, 3, and 9. Orange arrows highlight the increase or decrease of the studied markers through the total length of the experiments.

**Figure S16.** Day 3 analysis of T-cell activation, exhaustion, and phenotype for cells cultured with HCMs or TransAct. Heat map with the different markers studied showing % of the cell population that is positive. Increase in % of the population is represented by a changing gradient of dark purple (0%) to light yellow (100%). CD69: early-stage activation, CD25: late-stage activation, Tim3: exhaustion, CD62L+ CD45RO-: naïve T-cells, CD62L-CD45RO-: effector T-cells, CD62L- CD45RO+: effector memory T-cells and CD62L+ CD45RO+: central memory T-cells.

**Figure S17.** Day 9 analysis of T-cell activation, exhaustion, and phenotype for cells cultured with HCMs or TransAct. Heat map with the different markers studied showing % of the cell population that is positive. Increase in % of the population is represented by a changing gradient of dark purple (0%) to light yellow (100%). CD69: early-stage activation, CD25: late-stage activation, Tim3: exhaustion, CD62L+ CD45RO-: naïve T-cells, CD62L-CD45RO-: effector T-cells, CD62L- CD45RO+: effector memory T-cells and CD62L+ CD45RO+: central memory T-cells.

**Figure S18.** Percentage of the population A) that was transduced after 24h or B) exhibiting Tim3 exhaustion after 24h for the full population and transduced population. Color schemes: TransAct (light blue), CD3+CD28 (black), All 3:1 (dark purple), and All 1:1 (light purple). Four different donors with n≥3 for each donor were used as representative sample population. Standard deviation is shown in all the graphs.

**Figure S19.** Transduction at MOI of 1 (stripes) and 5 (solid) for T-cells activated with TransAct (light blue) and All 3:1 HCM (dark purple), with all the donors averaged. Four different donors with n≥3 for each donor were used as representative sample population. Statistical significance was determined using two ways ANOVA with post-Tukey test, and standard deviation is shown in all the graphs, ns= non-significant.

**Figure S20.** A) % cytolysis at 24, 48, and 72 h comparing CAR T cells generated by activation with All 3:1 and TransAct and transduction with CD19 virus. B) % cytolysis over time with measurements at every hour for 72h, where cytolysis increase over time can be observed. TransAct represented with light blue and All 3:1 represented with dark purple. One donor with n≥3 was used as representative killing potential. Statistical significance was determined using T-test finding no significant differences.
